# 3D Power Line Extraction from Multiple Aerial Images

**DOI:** 10.3390/s17102244

**Published:** 2017-09-29

**Authors:** Jaehong Oh, Changno Lee

**Affiliations:** 1 Department of Civil Engineering, Chonnam National University, Gwangju 61186, Korea; ojh@jnu.ac.kr; 2 Department of Civil Engineering, Seoul National University of Science and Technology, Seoul 01811, Korea

**Keywords:** power line, drone, photogrammetry, 3D mapping, cubic grid points

## Abstract

Power lines are cables that carry electrical power from a power plant to an electrical substation. They must be connected between the tower structures in such a way that ensures minimum tension and sufficient clearance from the ground. Power lines can stretch and sag with the changing weather, eventually exceeding the planned tolerances. The excessive sags can then cause serious accidents, while hindering the durability of the power lines. We used photogrammetric techniques with a low-cost drone to achieve efficient 3D mapping of power lines that are often difficult to approach. Unlike the conventional image-to-object space approach, we used the object-to-image space approach using cubic grid points. We processed four strips of aerial images to automatically extract the power line points in the object space. Experimental results showed that the approach could successfully extract the positions of the power line points for power line generation and sag measurement with the elevation accuracy of a few centimeters.

## 1. Introduction

Power line cables supported by transmission towers carry electrical power from a power plant to an electrical substation. Tower structures have been widely used to transmit high voltage current and more than 40,000 towers have been constructed in South Korea [1]. These towers and power line cables are constructed considering safety, as well as economic feasibility. The interval between the towers should be sufficiently long to minimize the number of towers, but sufficiently short to ensure minimum tension while providing safe clearance from the ground.

The periodic 3D mapping of power lines is critical for power line maintenance. Power lines are mostly fabricated of ACSR (aluminum-conductor steel-reinforced cable) and are constructed with a proper dip by loosening the cable. The power line dip is defined as the difference in level between the points of support and the lowest point on the line. Maintaining the proper dip is important because, while a large dip decreases the tension for better safety, it also decreases the clearance from the ground. When power lines stretch and sag with the changing weather, they can eventually exceed the planned tolerances. Excessive sagging can cause serious accidents and degrade the durability of the power lines. In addition, the sagging cables can sway in strong winds, touching adjacent topographic features.

Many remotely-sensed data products, such as SAR (synthetic aperture radar), thermal sensor, LiDAR (light detection and ranging), land-based mobile mapping data, and UAV (unmanned aerial vehicle), have been studied for power line surveys [2]. Limiting the scope of application to the power line extraction, LiDAR systems on airplanes and helicopters have been used to create and extract point clouds of power lines [3,4,5]. While the systems offer the sufficient elevation accuracy within ±15 cm, the operation of the system is often limited by high cost and local conditions, such as flight restrictions. Recently, compact and lightweight LiDAR sensors have been introduced to the market, although their performance is limited in terms of scan speed and measurement rate. Lately, drone systems with photogrammetric capability have strong potential for data acquisition and the 3D mapping of facilities in small areas. Drone systems, such as quadcopters, have gained popularity because of their agility, low lost, and hardware compatibility. A commercial low-cost drone comprises a 4K camera with a three-axis gimbal for photogrammetric use and offers autonomous flight for easy and safe data acquisition. Kuhnert and Kuhnert [6] presented mini and micro drones including a laser scanner for 3D monitoring of high voltage power lines. Liu et al. [7] established a flying robot mission-planning system for power line inspection and Ceron et al. [8] generated a process for navigation based in tower detection. Some studies have been carried out in which drones are used for power line inspections [9,10]. However, in these studies, the aerial images had only limited uses of manual inspection, orthophoto generation, and color information for 3D point cloud acquired from LiDAR. It is worth mentioning that low-cost drone systems have limitations of the battery capacity that only allows 10–20 min of flight. Additinoally, the systems may not be operated under severe weather conditions, such as strong wind.

Regarding the automated power line extraction in 2D aerial image space, Li et al. [11] presented a method including a pulse coupled neural filter and Hough transform to detect power lines on image. Sharma et al. [12] proposed an adaptive thresholding with a morphological filter to detect power lines on oblique video images. They reported less than 3% false positives. Yang et al. [13] used the Hough transform and a fuzzy C-means clustering to tolerate noise from complicated backgrounds for power line detection from UAV video images.

Some studies utilized 3D point cloud extraction of power lines from multiple aerial images. Yan et al. [14] used aerial images from an aerial digital camera onboard a helicopter for the power line extraction. A Radon transform and the Kalman filter were used to extract and connect the line segments into an entire line. The results were statistically analyzed for the extraction length in the image space. Zhang et al. [15] used a fixed wing UAV for the power line inspection proposing a semi-patch matching algorithm based on an epipolar geometry of stereo images. They reported the experimental results of the elevation accuracy of 0.5 m. Jozkow et al. [16] carried out the dense image matching for point cloud generation and filtering for the 3D modeling of power lines that they reported a fitting accuracy of 5–9 cm. These approaches follow the conventional image-to-object space approach that is comprised of line detection, image matching, and 3D reconstruction.

In this study, we proposed the object-to-image space approach using cubic grid points for 3D power line mapping. The aim of the study was to derive 3D power line point cloud from multiple aerial images acquired using a low-cost drone. In the method, each aerial image provides 2D power line primitives that are used to filter the dense 3D cubic grid points generated around the power line on the ground. The relation between the image and object spaces is established by performing accurate bundle adjustment.

The paper is structured as follows: In Section 2, the proposed method is explained and the bundle adjustment, line segment extraction, and cubic grid generation and filtering are discussed. The experimental results are presented in Section 3, followed by conclusions in Section 4.

## 2. Method

**Figure 1:**
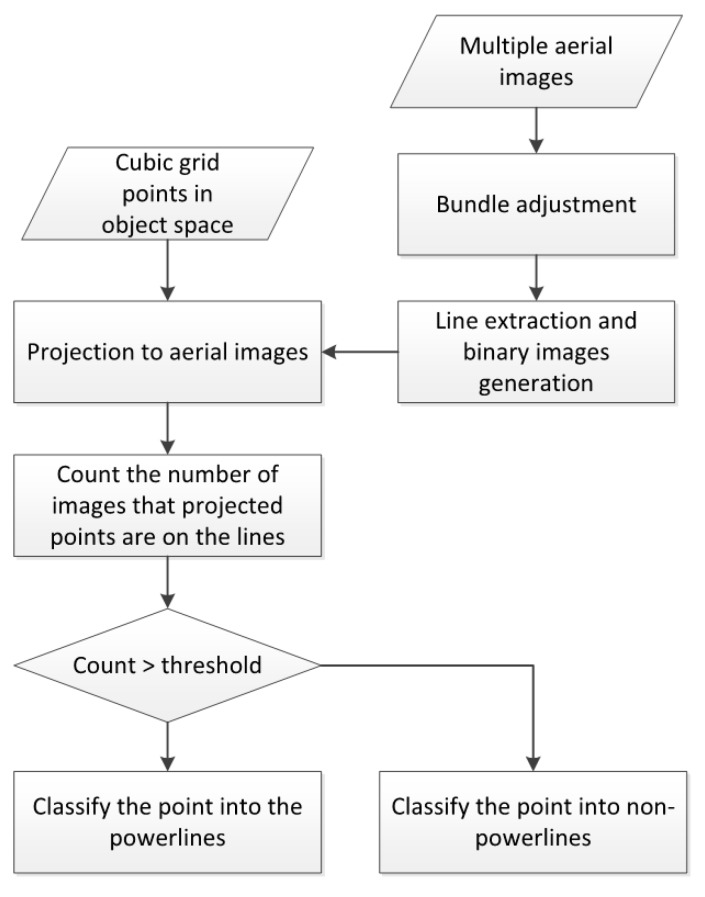
Flowchart of the power line extraction.

A flowchart of the proposed method is presented in Figure 1. The input data are multiple aerial images acquired using a low-cost drone. The data are preprocessed with the bundle adjustment to accurately estimate the IOPs (interior orientation parameters) of the camera and EOPs (exterior orientation parameters) of each image. Each image is processed for 2D power line segments and binary images are generated as the results. The pixel values in the binary images are all 1, if they are located on line segments. In the object space, cubic grid points are generated around the power lines and all points are projected into each image space using the estimated IOPs and EOPs. We then count the number of images in which the pixel value of the projected location is one (i.e., line segment). Finally, if the counted number is larger than an established threshold, we classify the point in the power line point group.

### 2.1. Image Acquisition and Bundle Adjustment

Even low-cost drones provide functions for the autonomous flight mission. The flight plan including the flying height above ground and overlaps can be easily set up in the mobile devices. For power lines mapping, the safety and GSD (ground sampling distance) of images are taken into account for planning the flying height. The GSD can be estimated using the flying height, the focal length, and the pixel pitch in Equation (1). The information about the camera and sensor can be found in the specification from manufacturers. Typical overlaps of the aerial image acquisition range from 60% to 80% but higher overlaps are preferred for the facility mapping in a small region because they provide greater redundancy.

(1) $$powerline\;GSD=(pixel\;pitch)\times \frac{\left(flying\;height\right)}{\left(focal\;length\right)}$$

The acquired images are tagged with the position and attitude information from installed GNSS (global navigation satellite system) and IMU (inertial measurement unit). However, the reliability of the system in a low-cost drone is not high so that the images need to be processed with the bundle adjustment for accurate IOPs and EOPs estimation [17].

### 2.2. Line Extraction in Image Space

**Figure 2:**
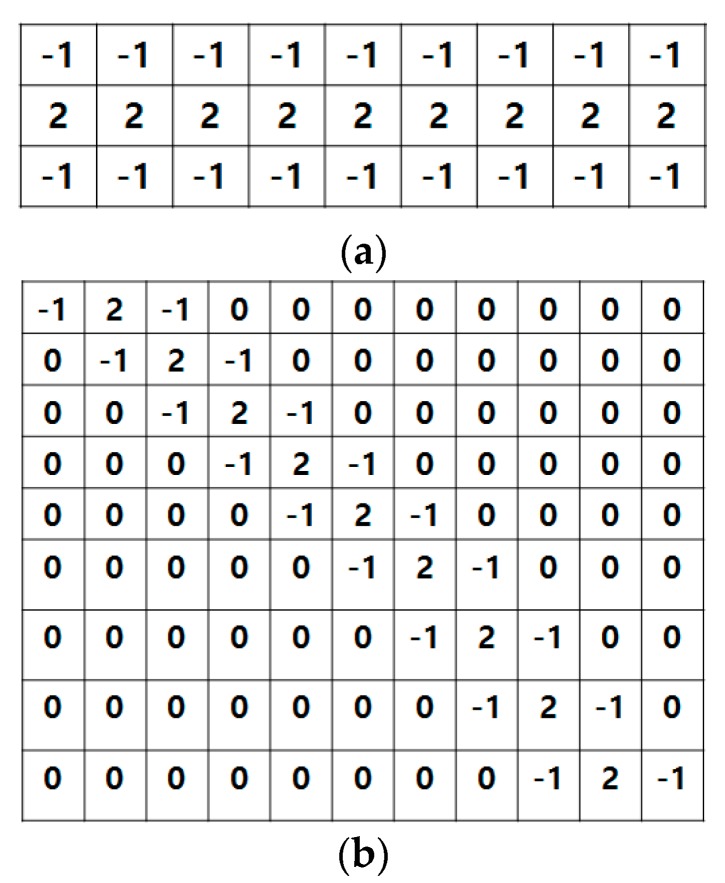
Filters to extract horizontal (**a**) and diagonal (**b**) lines in the image space.

We carried out the power line pixel extraction from each aerial image using a simple template as shown in Figure 2 because power lines on aerial images are close to a straight line with a very small curvature. In the flight mission, the direction of drone flight can be set across the power lines which results in the imaged power lines appearing horizontal. For these power lines, the horizontally-extended filter in Figure 2a has been designed to constrain the noise with −1 in the first and third lines and 2 in the middle [14]. For the vertical and any other diagonal directions, filters with different angles of 45°, 90°, and 135° can be used as one example is depicted in Figure 2b.

Applying the filter enhances the power lines that have strong responses and suppresses the backgrounds. Thus, the candidate pixels of power lines are extracted.

The candidate pixels after the extraction are connected as line segments using connected-component labeling [18]. In this study we analyzed the 4-neighbors connectivity from a pixel, i.e., (x + 1, y), (x − 1, y), (x, y + 1), and (x, y − 1). The line segments with a threshold longer than that given are only selected as meaningful power line segments in the image space.

### 2.3. Cubic Grid Points Generation in Object Space and Power Line Points Selection

**Figure 3:**
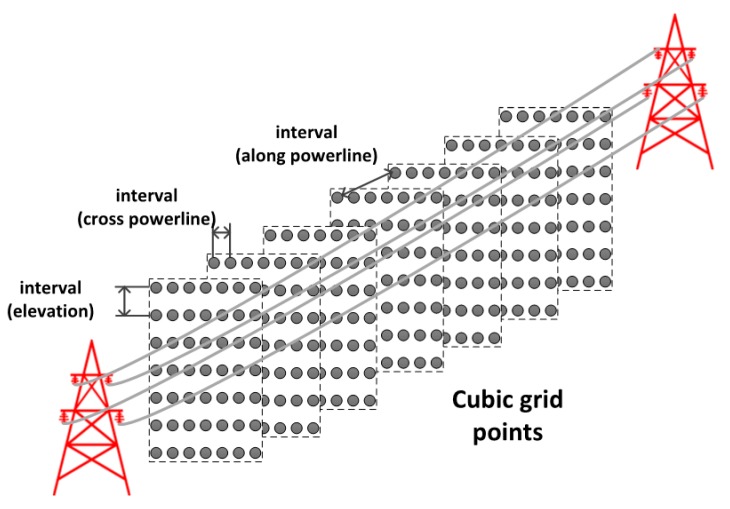
Generation of cubic grid points around the power lines in the object space (3D).

The cubic grid points are generated around the targeted power lines in the 3D object space as in Figure 3. The horizontal boundary of the grid can be roughly set on a map and the spacing between points is selected taking into account the GSD of the acquired images. For example, the point interval crossing the power lines should be set at around the GSD of the images in order to avoid skipping over the line information while the interval along the power lines can be very loosely set to be larger than GSD. The elevation interval can be set to around the GSD for precise elevation information. As depicted in Figure 3, the cubic grid points are a series of planes (sections) along the power line comprising of grid points. Some of the grid points are located at the power lines and the other points are not. Therefore, we would like to select those points at the power lines using the multiple image information.

**Figure 4:**
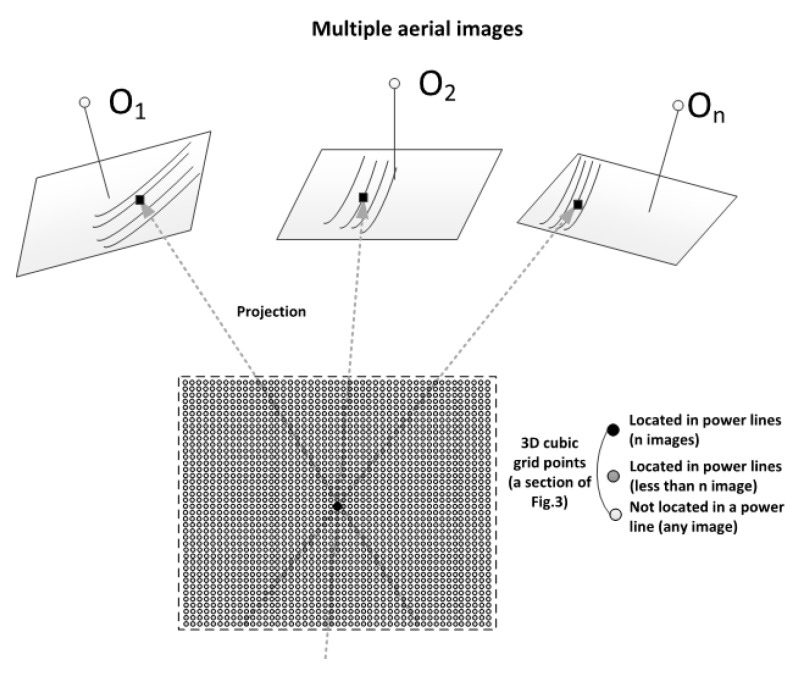
Projection of cubic grid points (3D) into multiple images (2D) for power line point selection.

Given a cubic grid point, the grid point is projected to the multiple aerial images. In Figure 4a grid point is classified depending on the number of images on which the projected pixel is a power line segment. If the number of images is larger than a threshold, we classify the cubic grid point into the power line point. This process is iterated for all cubic grid points.

The object to image projection of a grid point is carried out using the well-known collinearity equation (Equation (2)). The equation includes EOPs and IOPs. EOPs are the positions and attitudes of the camera at the moment of exposure. IOPs are the parameters of the camera, such as the focal length and distortions.

(2) $$\left[\begin{array}{l} x-x_{0}+\Delta x\\ y-y_{0}+\Delta y\\ -f \end{array}\right]=\lambda M\left[\begin{array}{c} X-X_{L}\\ Y-Y_{L}\\ Z-Z_{L} \end{array}\right]$$

where:
x,y:coordinates of an image point in the photo coordinate system (conventionally, x is along the flight direction);f:focal length;M:rotation matrix from the ground coordinate system to the camera coordinate system, which is determined by the attitude of the camera at the moment of exposure;X,Y,Z:coordinates of a ground point in the ground coordinate system;$X_{L},Y_{L},Z_{L}$:coordinates of camera position at the moment of exposure in the ground coordinate system;$x_{0},y_{0}$:principal point offsets;Δx,Δy:camera distortion;λ:scale factor (photo scale).

The rotation matrix M is constructed using three sequential rotations: roll (ω), pitch (ϕ), and yaw (κ), as given in Equation (3):

(3) $$M=M_{\kappa }^{}M_{\phi }^{}M_{\omega }^{}$$

### 2.4. Power Line Generation

**Figure 5:**
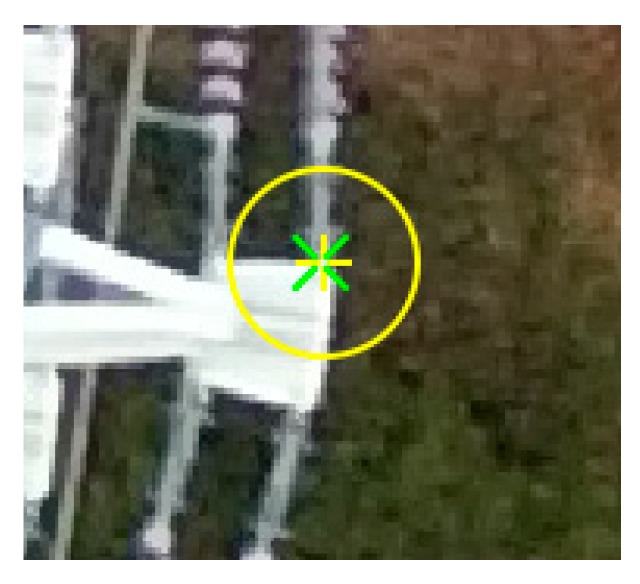
Example of the point of support at the tower structure.

After the power line points are selected from the cubic grid points, we generate the power line by interpolating the point cloud using the parabola equation. This equation requires the points of supports at the tower structures, which can be manually extracted as shown in Figure 5. Let the coordinates of the points be $\left(X_{1},Y_{1}\right),\left(X_{2},Y_{2}\right)$. The variable of the parabola equation is the distance D from one point of the support $\left(X_{1},Y_{1}\right)$ to a point along the power line $\left(X_{i},Y_{i}\right)$. The coefficients of the equation a,b,c can be estimated using the least square with the fixed constraints of the point of supports.

(4) $$\begin{array}{l} Z_{i}=aD_{i}^{2}+bD_{i}+c\\ D_{i}=\sqrt{{\left(X_{i}-X_{1}\right)}^{2}+{\left(Y_{i}-Y_{1}\right)}^{2}} \end{array}$$

## 3. Experiment

### 3.1. Data Acquisition

**Figure 6:**
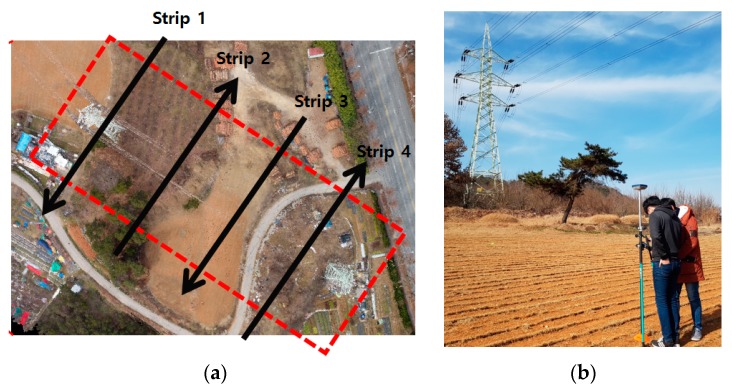
Test site (**a**) and GNSS surveying (**b**).

The test site is in Gwangju City of Korea, as shown in Figure 6a. This site was selected for the test because it has an easy approach and is safe for flying the drone. The targeted power lines carry 154 kV electric power and the span between the double-circuit power towers is about 100 m, which is relatively shorter than the typical spans of 300–400 m. The height of each tower is about 40 m above the ground. The tower has three cross arms, where two power lines are connected at each end such that a total of 12 power lines are connected via the tower. In addition, a guard wire is placed on top of the towers.

We used a low-cost drone, the Phantom 4 (DJI), the specifications of which are shown in Table 1. The focal length of the installed camera is 3.6 mm and the camera produces 12.4 megapixel images. While lower altitudes can produce better spatial resolution, care must be taken to avoid collision with the structures. We set the flying altitude as 80 m above the ground, considering the safety height above the towers, which is 40–50 m above the power lines. The GSD at power line elevation was estimated to be 1.75–2.2 cm. While the estimated power line GSD is slightly larger than the diameter of the power line (about 1.5 cm), the lines are observable in the images due to their continuity along the line. The images were acquired with 80% overlap and side laps for multiple image processing. We also carried out a network RTK (real-time kinematic)–based GNSS survey (Figure 6b) for the bundle adjustment and its accuracy assessment.

**Figure 7:**
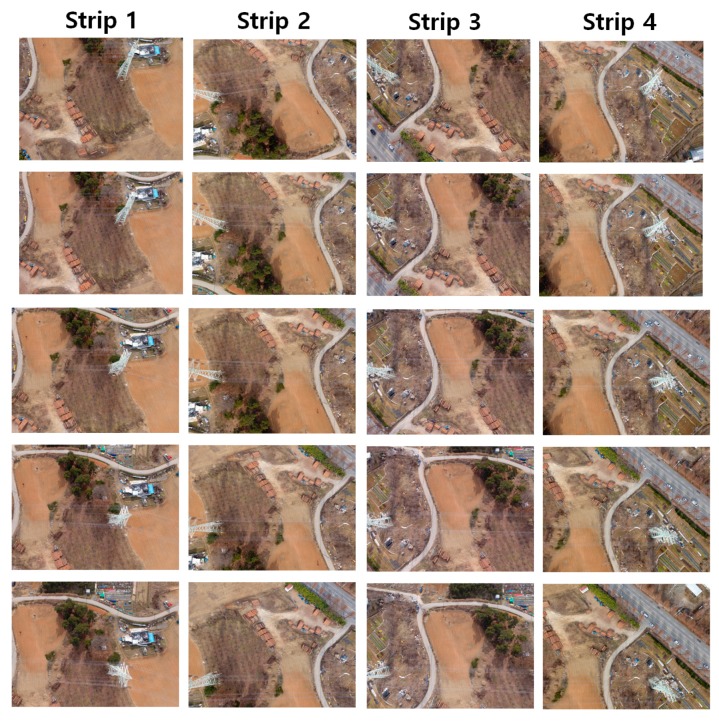
The four acquired strips of aerial images.

During the flight mission, four strips of images crossing the power lines were produced, as shown in Figure 7. Each strip comprises five images, where the tower structures and power lines can be observed. The power lines are shown along the horizontal direction.

**Figure 8:**
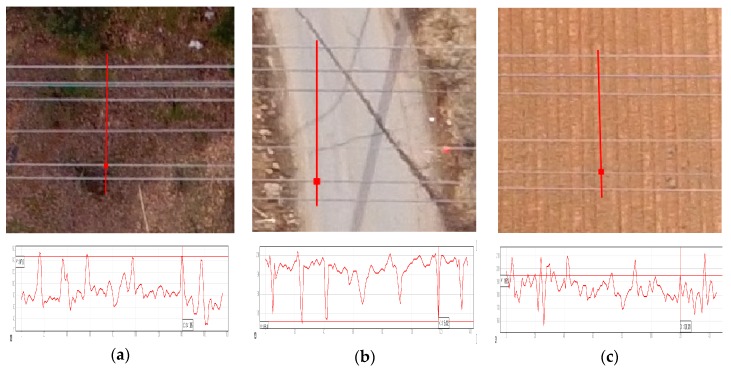
Power line image samples for different backgrounds.

Though the diameter of all ASCR power lines is around 1.5 cm, the power lines are shown at 2–3 pixels because of their linear pattern and the contrast with the backgrounds, as shown in Figure 8. The figures show three power line sample images with different intensity profiles. The contrasts observed of power lines against backgrounds are good, moderate, and poor for the green vegetation (Figure 8a), road (Figure 8b), and bare soil (Figure 8c), respectively.

### 3.2. Bundle Adjustment with Camera Calibration

We used Pix4Dmapper Pro for the bundle adjustment. The software uses the position and attitude information from installed GNSS and IMU sensors for the initial approximation. For the bundle adjustment, over 20,000 tie points per image and a total of 12 GCPs are utilized. The interior and exterior orientation parameters were adjusted together, i.e., on-the-fly self-calibration. Table 2 shows the precision of EOPs from the adjustment. The camera positions at the moment of exposure are estimated with the precision of 1–3 cm and the attitudes are estimated with the precision of 0.004–0.011°.

Table 3 shows the errors of the adjustment in RMSE (root mean square error) for 12 GCPs and five check points GNSS-surveyed at the ground. The horizontal errors are in the range of 1–2 cm, which is less than the estimated ground GSD which is 3.24 cm, while the elevation errors are in the range of 2.3–5 cm, which is around the ground GSD. GCP errors are generally smaller than those at check points, but in this experiment the results seem to be the other way around. However, the differences between the errors are not at the significant level in horizontal direction (X, Y) considering the ground GSD. The elevation difference between them is relatively large, but it is also at the uncertainty level. Note that the low-cost camera has much lens aberration for blurred ground targets in the image and the uncertainty level is higher than the usual aerial photogrammetric process. When we checked the quality in the image space by reprojecting the ground points onto the images, the residual and errors were less than one pixel.

### 3.3. Line Extraction

**Figure 9:**
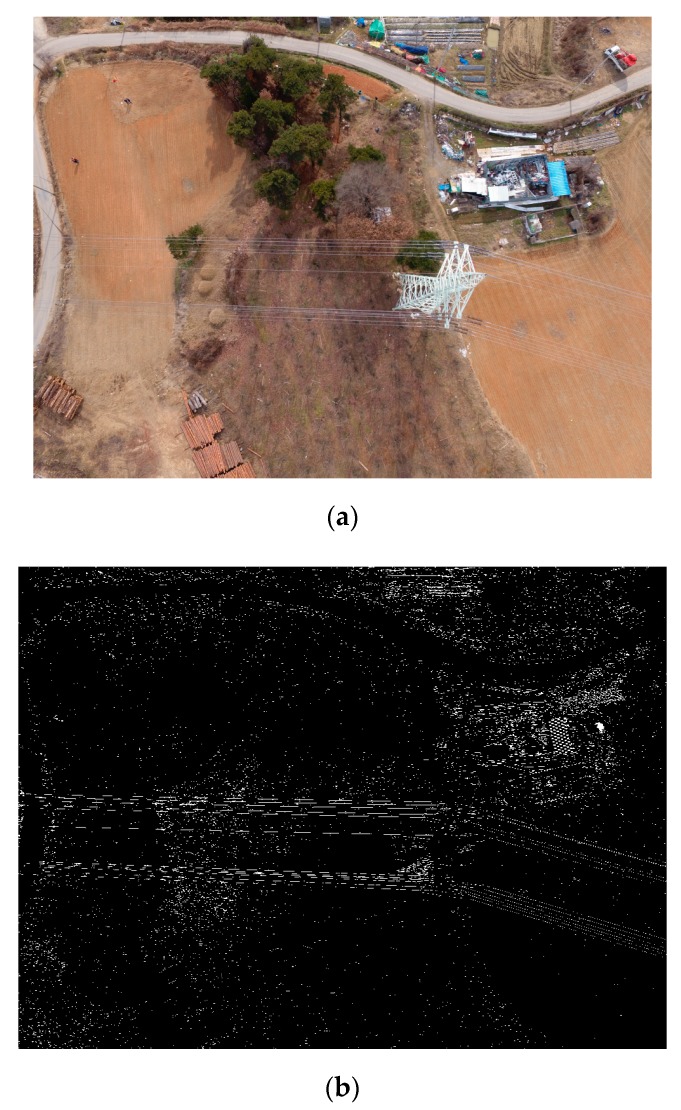

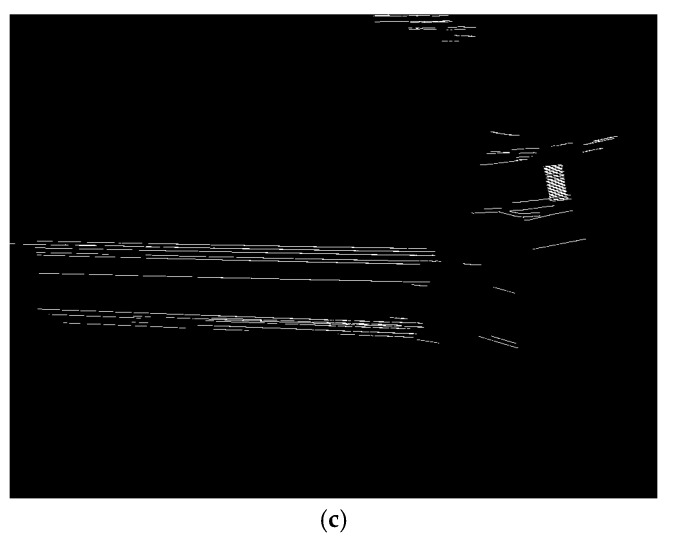
Example of horizontal line extraction.

We used a 3 × 9 horizontal line extraction filter, since most power lines run along the horizontal direction of the images. This filter generated noisy line components over the image space and the connectivity analysis was then used to label the components. To suppress noise, we selected only those line components with a length greater than 100 pixels. Figure 9a,b show an example of the line extraction results for one image in strip 1. The labeling and the removal of the short line components produced less noisy line components shown in Figure 9c.

### 3.4. Cubic Grid Points Processing

We set a region around the power lines in a map to generate cubic grid points. The red box in Figure 6a shows an example of the region. The elevation range was set using the prior information about the power lines that range from 60 m to 90 m above the earth ellipsoid. With establishing the intervals between grid points we could generate the cubic grid points in the 3D object space. Note that the intervals should not be too large because the points may not be on the individual power lines in the object space.

**Figure 10:**
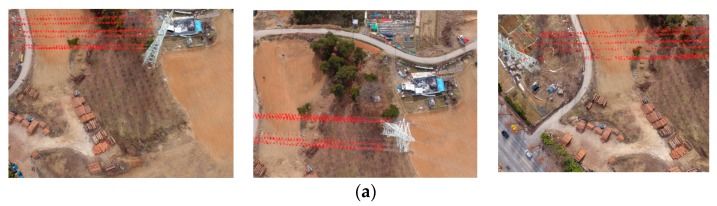

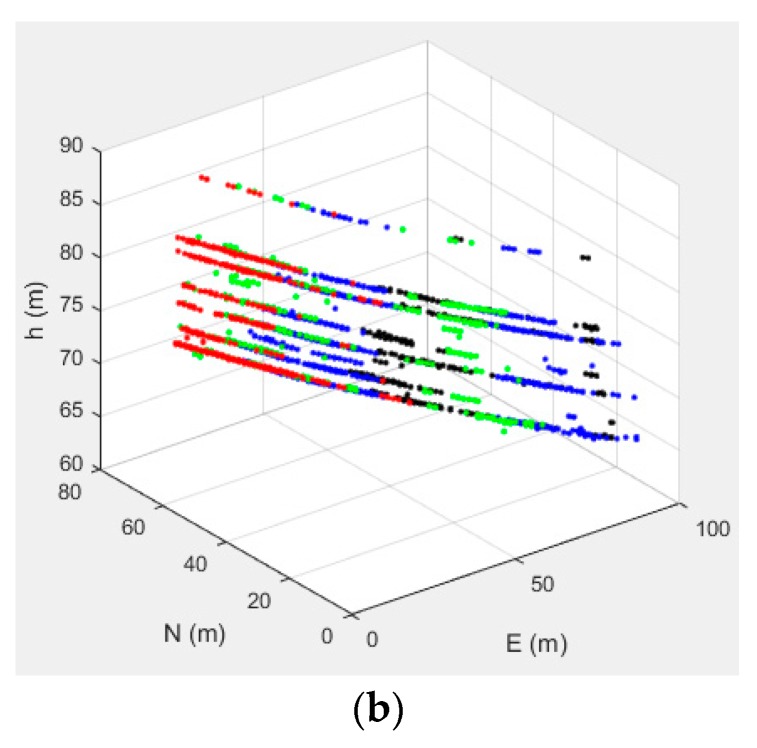
Generated power line points for the less dense interval case with threshold 4 (red, green, blue, and black for Strips 1, 2, 3, and 4, respectively).

In this experiment, the cubic grid points were generated using two cases: less dense and dense. For the less dense case, we used intervals of 1 m, 5 cm, and 5 cm for along the power lines, across the power lines, and the elevation directions, respectively. For the dense case, we used intervals of 0.3 m, 2 cm, and 2 cm, for along the power lines, across the power lines, and the elevation directions, respectively. We then iterated the projection of the cubic grid points into each image strip which consists of five aerial images. In each strip, the number of images is counted when the projected location is on a line segment. If the number of images is larger than the established threshold, the point is classified in the power line point group. We carried out experiments for two threshold cases of four and five images. Figure 10 show an example of extracted power line points for the less dense case with a threshold of 4. Figure 10a shows the extracted points in some sample images. In Figure 10b, different colors are used to 3D plot the different strips. At the top of the 3D plot, the guard wire is shown connecting the top of the towers and the 12 power lines shown underneath. While six lines are shown due to the display scale, they represent 12 lines. Note that two power lines are installed at each side of one cross arm for a total of 12 lines at three cross arms.

### 3.5. Validation

**Figure 11:**
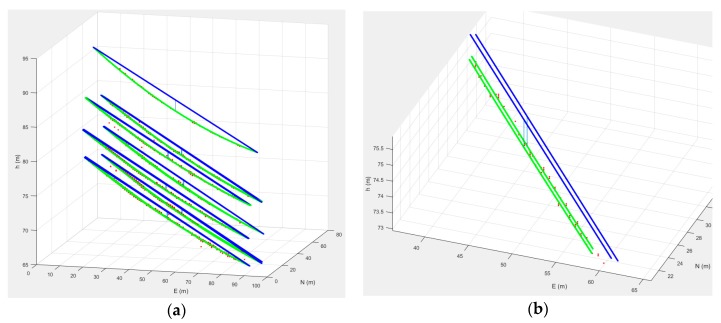
Interpolation of the power lines.

We interpolated the power line points using the parabola equation, connecting the points of supports at the tower structures. Figure 11a shows the results where the blue lines represent straight lines connecting the points of support at the power towers (we call these reference lines), and the green lines represent the interpolated power lines. The figure also shows the point of the maximum sag showing the difference between the straight lines and the power lines. Figure 11b shows a magnified view of the straight lines and power lines.

Table 4 shows the interpolation residual for each power line and the guard wire. Most cases showed the residuals range from 4–16 cm. The guard wire only shows 0.4 cm for the less dense case with a threshold of 5, because few points were extracted and they did not provide sufficient redundancy for the interpolation. When we switched the threshold from four to five images, the residual decreased, providing stricter point filtering. The residuals of the less dense and dense cases do not show large differences considering the power line GSD of a few centimeters.

Table 5 shows the sag measurements for each power line computed by measuring the maximum difference in elevation between the reference line (straight line connecting the points of support) and the interpolated power line. The measured sags range 1–2 m for the short span between the towers. These measurements are quite small considering that the power lines with much larger spans show sags from a few meters up to more than ten meters. All experiment cases showed consistent estimations with a standard deviation of 1–4 cm.

Table 6 shows the elevation differences between the derived power lines and the ground truth data which were measured at the ground using a reflectorless total station instrument for line #1 and #12. The differences are about 3.9 cm and 6.2 cm for line #1 and #12, respectively, which show similar levels of accuracy as those in the results of the bundle adjustment. While the differences can include errors from the bundle adjustment, line extractions, and thickness of the line, the major error source should be the bundle adjustment because the line extraction contributes 1–2 pixels if there is no outlier in the line extraction process. It is mentionable that the terrestrial measurement was difficult because the measurement beam did not often return due to the thinness of the power lines. Additionally, because line #12 is located further from the surveying instrument than that of line #1, the quality of the reference is less reliable than that of line #1.

The resultant accuracy is acceptable for power line monitoring, which typical requires the elevation accuracy of 20 cm. We cannot directly compare the results to that of other studies because the system, the operation condition, and sites are different. But the result is decent that LiDAR systems show elevation accuracy within ±15 cm and previous studies on using aerial images reported the 3D power line extraction accuracy of 0.5 m referring to the ground truth data [15] and the fitting accuracy of 5–9 cm not using the ground truth [16].

## 4. Conclusions

We used photogrammetric techniques with a low-cost drone to achieve efficient 3D mapping of power lines that are often difficult to approach. Unlike the conventional image-to-object space approach that extracts image features, performs image matching, and reconstructs 3D coordinates, we used the object-to-image space approach using cubic grid points. We processed four strips of aerial images for automatic extraction of the power line points in the object space. We observed that changing the cubic grid point density did not significantly affect the sag measurement of the power lines. When we increase the threshold from four to five images, the population of extracted power line points was reduced but the line interpolation precision increased. Finally, experimental results showed that the approach successfully extracted power line points for the power line generation and the sag measurement at the accuracy of a few centimeters. For future study, more experiments are required for power lines in a variety of environments and backgrounds, such as snow. It will also include the 3D topographic mapping and analysis for near-zones of electric-power facilities using visible and multi-spectral sensors to extract hazardous topography.

## Figures and Tables

**Table 1:** Specifications of the drone and camera used.

Drone—Phantom 4		Camera	
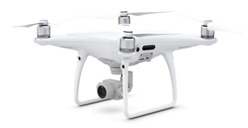		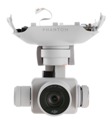	
Weight	1380 g	Focal length	3.6 mm
Max speed	20 m/s	Pixel pitch	0.00158 mm
Flight time	28 mins	FOV	94 deg
GNSS	GPS GLONASS	Sensor size	12.4 M (4000 × 3000)

**Table 2:** Precision of the camera position and attitudes.

	X [cm]	Y [cm]	Z [cm]	Roll (ω) [deg]	Pitch (ϕ) [°]	Yaw (κ) [°]
Mean	1.7	1.4	3.1	0.011	0.011	0.004

**Table 3:** Bundle adjustment errors in RMSE.

	GCPs	Check Points
X	1.8 cm	1.4 cm
Y	0.9 cm	1.4 cm
Z	5.0 cm	2.3 cm
Image space	0.89 pixels	0.95 pixels

**Table 4:** Power line interpolation residuals in RMSE (cm).

Line #	Less Dense		Dense	
Threshold (# of images)	4	5	4	5
Guard wire	9.1	0.4	8.0	7.8
Line 1	7.7	5.2	7.6	5.0
Line 2	16.2	5.7	15.6	5.3
Line 3	8.9	5.7	9.2	6.2
Line 4	7.4	4.2	8.1	6.1
Line 5	9.1	6.4	9.0	5.5
Line 6	11.1	6.4	10.4	7.1
Line 7	11.3	4.1	11.2	4.7
Line 8	9.3	3.1	11.7	4.4
Line 9	8.7	4.9	13.2	5.2
Line 10	10.8	3.4	15.2	8.9
Line 11	16.5	4.7	15.4	6.5
Line 12	11.2	5.7	10.8	8.1
Mean(abs)	10.56	4.61	11.18	6.22

**Table 5:** Measured sags (m).

Line #	Less Dense		Dense		Mean	Std
Threshold (# of images)	4	5	4	5	-	-
Guard wire	1.62	1.69	1.59	1.63	1.63	0.04
Line 1	1.00	1.02	1.00	1.01	1.01	0.01
Line 2	1.11	1.12	1.09	1.08	1.10	0.02
Line 3	0.87	0.91	0.88	0.89	0.89	0.01
Line 4	0.94	0.99	0.95	0.97	0.96	0.02
Line 5	0.95	0.96	0.95	0.96	0.96	0.01
Line 6	0.99	1.01	0.97	0.99	0.99	0.01
Line 7	0.95	1.00	0.95	1.00	0.98	0.03
Line 8	1.03	1.06	1.04	1.05	1.05	0.01
Line 9	0.99	0.97	0.97	0.95	0.97	0.01
Line 10	0.91	0.99	0.91	0.93	0.94	0.03
Line 11	0.86	0.89	0.86	0.88	0.87	0.01
Line 12	0.87	0.93	0.88	0.89	0.89	0.02

**Table 6:** Elevation differences between the derived power lines and the ground truth in RMSE (cm).

Line #	Less Dense		Dense		Mean	Std
Threshold (# of images)	4	5	4	5	-	-
Line 1	4.0	3.6	4.1	3.7	3.9	0.2
Line 12	6.9	5.3	6.5	6.1	6.2	0.6
